# Integrated Metagenomics/Metaproteomics Reveals Human Host-Microbiota Signatures of Crohn's Disease

**DOI:** 10.1371/journal.pone.0049138

**Published:** 2012-11-28

**Authors:** Alison R. Erickson, Brandi L. Cantarel, Regina Lamendella, Youssef Darzi, Emmanuel F. Mongodin, Chongle Pan, Manesh Shah, Jonas Halfvarson, Curt Tysk, Bernard Henrissat, Jeroen Raes, Nathan C. Verberkmoes, Claire M. Fraser, Robert L. Hettich, Janet K. Jansson

**Affiliations:** 1 Chemical Science Division, Oak Ridge National Laboratory, Oak Ridge, Tennessee, United States of America; 2 Graduate School of Genome Science and Technology, University of Tennessee, Knoxville, Tennessee, United States of America; 3 Institute for Genome Sciences, University of Maryland School of Medicine, Baltimore, Maryland, United States of America; 4 Department of Ecology, Earth Sciences Division, Lawrence Berkeley National Laboratory, Berkeley, California, United States of America; 5 Bioinformatics and Eco-Systems Biology lab, Department of Structural Biology, Vrije Universiteit Brussel, Brussels, Belgium; 6 Research Group of Microbiology (MICR), Faculty of Sciences and Bioengineering Sciences, Vrije Universiteit Brussel, Brussels, Belgium; 7 Department of Internal Medicine, Division of Gastroenterology, Örebro University Hospital and School of Health and Medical Sciences, Örebro University, Örebro, Sweden; 8 Architecture et Fonction des Macromolécules Biologiques, UMR6098, Centre national de la recherche scientifique, Universités Aix-Marseille I & II, Marseille, France; University of Illinois, United States of America

## Abstract

Crohn's disease (CD) is an inflammatory bowel disease of complex etiology, although dysbiosis of the gut microbiota has been implicated in chronic immune-mediated inflammation associated with CD. Here we combined shotgun metagenomic and metaproteomic approaches to identify potential functional signatures of CD in stool samples from six twin pairs that were either healthy, or that had CD in the ileum (ICD) or colon (CCD). Integration of these omics approaches revealed several genes, proteins, and pathways that primarily differentiated ICD from healthy subjects, including depletion of many proteins in ICD. In addition, the ICD phenotype was associated with alterations in bacterial carbohydrate metabolism, bacterial-host interactions, as well as human host-secreted enzymes. This eco-systems biology approach underscores the link between the gut microbiota and functional alterations in the pathophysiology of Crohn's disease and aids in identification of novel diagnostic targets and disease specific biomarkers.

## Introduction

Humans live in close association with communities of microorganisms (the human microbiota) that inhabit every exposed surface and cavity in the body [1]. The collective genetic information of the human microbiota represents a second genome, the human microbiome, currently the focus of intense international sequencing and research efforts [2]–[7]. To date the main focus has been on using high throughput sequencing to determine the composition of the human microbiome in healthy individuals (e.g. characterization of the human microbiome across different body sites [5] and across different ages and geographic areas [7]. Several of these studies have found a large variation in the gut microbial community composition between individuals, but considerable functional redundancy [5], [8].

The next step is to determine how the human microbiome varies with disease. As part of a demonstration project funded through the NIH Human Microbiome Project (HMP) we have focused on the impact of the inflammatory bowel disease (IBD), Crohn's disease on the gut microbiota. Although most human host-microbe associations are beneficial, several studies using both culture-dependent and molecular approaches have suggested that there is a dysbiosis in the gut microbiota of patients with Crohn's disease (CD) compared to healthy subjects [9]–[13]. In the current study we specifically aimed to focus on functional differences in the gut that may account for the previously observed dysbiosis.

Although recent advances in DNA sequencing and proteomics technologies have opened the door to investigation of the structure and function of the gut microbiota without the necessity for cultivation, there have been very few efforts to date that have used a multi-“omics” approach to study the complex ecosystem in the human gut [14]. The ability to combine information about the *identities* of microbial community members (obtained from 16S rRNA gene-based measurements), *metabolic potential* (obtained from metagenome sequence data) and *expression* (obtained from metaproteome data) should enable exploration of the gut microbiota at multiple molecular levels simultaneously.

This study was focused on a subset of stool samples collected from a large Swedish twin cohort with inflammatory bowel disease (IBD) that was previously characterized with respect to their bacterial community composition by deep 16S rRNA pyrotag sequencing [15] and metabolite profiling [16]. Previous data indicated that healthy twin pairs had a similar gut microbiota, even when they had been living separately for decades [11], as also supported by other studies showing higher similarity between twins than between unrelated individuals [8]. By contrast, twin pairs in which one or both subjects had CD harbored very dissimilar gut microbial compositions [11]. This disparity of the gut microbiota was particularly striking for subjects with inflammation in the ileum (ileal CD, ICD) compared to healthy subjects [11], [15], [16] and was primarily characterized by the reduced abundance of several key beneficial members of the community, such as *Faecalibacterium prausnitzii*.

Here our aim was to further explore a subset of the same Swedish twin cohort for functions that were correlated to CD by applying non-targeted, shotgun metagenomics [17] and metaproteomics [18]. Although we know from our previous studies mentioned above that there were differences in the microbial communities and metabolite profiles between individuals with CD and healthy in this cohort, what is lacking is an understanding of the reasons for the differentiation of the samples in a functional context. By application of an eco-systems biology approach [19], here we were able to detect and directly correlate genes, proteins, and metabolic pathways for the first time in complex human gut samples. It was particularly valuable to include discordant twin pairs in the sample set, where one twin was diseased and one was healthy, thus representing some level of internal control of host genetics on the microbiome (Table S1 in Supporting Information S1).

The specific questions that we set out to address in this study were: (1) What genes are actually expressed as proteins in the gut and could play a functional role in the gut environment? (2) Are there specific genes and proteins that could help to explain the previously observed differentiation of the samples according to Crohn's disease etiology?

Shotgun metaproteomics is a relatively new technology in its' application to complex and highly diverse microbial communities, such as the human gut, and only recently have there been reports about protein compositions in the gut and from only a few healthy subjects [18], [20]–[23]. Therefore, in this study we deliberately selected samples that were previously well characterized and shown to significantly differ between healthy and CD for optimization of the methodology and to increase our chances of detecting proteins that could correlate to disease etiology. The sample cohort included one healthy twin pair, one colonic Crohn's (CCD) twin pair, two ICD concordant twin pairs and two ICD discordant twin pairs (Table S1 in Supporting Information S1). To perform these analyses we optimized a shotgun metaproteomics pipeline with matched metagenomes to obtain the most comprehensive coverage of human distal gut proteins to date.

## Results

### Data generation and sequence clustering

We generated shotgun metagenomic (Table S2 in Supporting Information S1) and shotgun mass spectrometry (MS)-based metaproteomic (Tables S3, S4, S5, S6) datasets from the same stool samples for direct comparisons. Metagenomic data were used to assess whole-community gene content and predicted functional capabilities of the gut microbiome, while metaproteomics was used to identify the measurable microbial and human proteins being expressed in the system.

### Assessment of expressed genes using metaproteomics

Metagenomic data does not reveal the identities and abundances of *expressed gene products* (proteins) under the conditions studied. Therefore, to directly address gene function and protein abundance, we performed database searches with tandem mass spectra (MS/MS) of peptides from the same samples collected via multi-dimensional liquid chromatography tandem mass spectrometry (2d-LC-MS/MS). These extensive MS/MS datasets were searched either against their corresponding matched metagenome (MM) (Table S2 in Supporting Information S1) or a representative set of 51 sequenced human microbial isolate reference genomes (HMRGs) (Table S7), each concatenated with the predicted human protein database (July 2007 release, NCBI). Although 51 reference genome sequences cannot capture all of the protein diversity within the human gut microbiota, we chose to select these as a minimal set of reference genomes based on genera that have been previously found in these samples [15]. By selecting only a subset of the larger bank of human isolate reference genomes that are being produced through the Human Microbiome Project [3], we aimed to reduce the sequence redundancy between species/strains that is a limitation of current MS database searching algorithms. While the isolate genomes chosen represent about 75% of the genera estimated by 16S analysis [15], the rest of the community is comprised of genera that represent less than 1% of the total community, or are unknown (Figure S1A in Supporting Information S1). The HMRGs provided complete gene sequences for many of the most abundant genera (Figure S1A in Supporting Information S1), in contrast to the MMs that had more fragmented sequence data from all of the taxa in the microbiota. However, relying solely on reference genomes for proteome identification limits the protein families identified to those in sequenced organisms, which is a small percentage of the total bacteria in the gut. To address the issue of gene redundancy between strains/species belonging to the same genera in the metagenome data, we developed a novel method for clustering of proteins from the MM datasets to provide a more robust method of assigning peptide-spectrum counts for relative quantification [23].

On average, a total of 1,250 (healthy), 850 (ICD), and 788 (CCD) orthologous protein clusters were identified with MM searches and 2,904 (healthy), 1,928 (ICD), and 2,241 (CCD) proteins using HMRG searches. Together, these data represent the largest metaproteome analysis of the human gut to date (Tables S3 and S4). To gauge the overlap in protein sequence coverage between the MM (read-based protein spectrum matches, PSMs) and HMRG databases, we compared the assigned, non-redundant spectra with high mass accuracy (±10ppm) with PSMs from both searches. Of the total spectra that have peptide assignments to microbial and human proteins, 64% and 33% of the PSMs were unique to the MM and HMRG databases, respectively (Figure S1B in Supporting Information S1). These results suggest that these databases are complimentary, each containing a large set of unique peptides that individually are a sampling of these very complex proteomes. This approach enabled us to take advantage of both MMs and HMRGs to identify disease-specific proteins associated with the human gut microbiota, including those with unknown function.

### General overview of metagenomic and metaproteomic datasets

**Figure 1:**
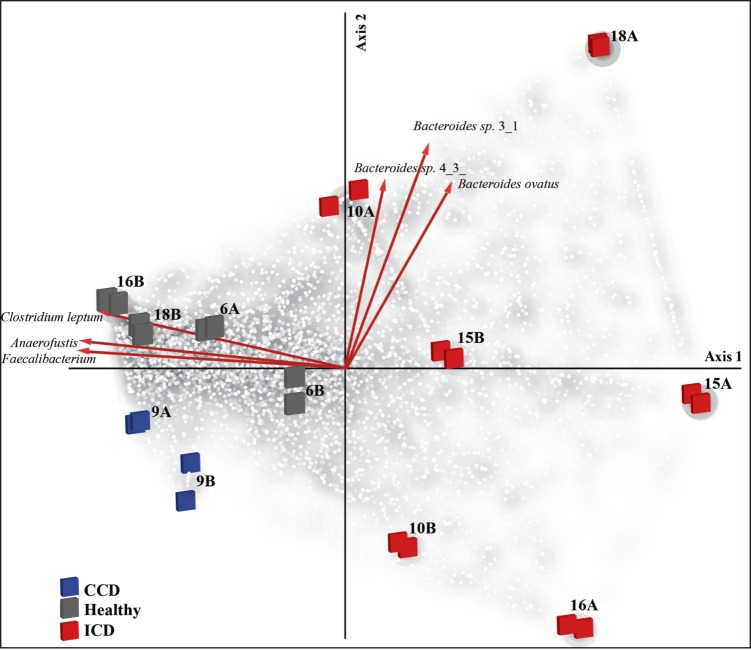
Clustering of distal gut metaproteomes according to disease. Non-metric multidimensional scaling (nMDS) of distal gut metaproteomes from CD twin cohort. The different colored square symbols represent the metaproteomic profiles for each sample (Blue  =  CCD, Grey  =  Healthy, Red  =  ICD). The numbers beside the symbols refer to the specific patient ID from Dicksved et al., 2008 (proteomes were run in technical duplicates). The axes are dimensionless: the coefficients of determination for the correlations between ordination distances and distances in the original n-dimensional space are. 472 and. 831 for Axis 1 and 2, respectively. A matrix of normalized spectral counts per protein (HMRG database search) from each duplicate metaproteome was imported into PCORD v5 software. nMDS was performed using the Bray-Curtis distance measure A three-dimensional solution was found after 119 iterations. The final stress for the nMDS was 6.47458. The white spots with grey shading correspond to individual proteins identified using HMRG database. Arrows indicate strength of correlation of specific bacterial strains to ordinated data. Pearson correlation coefficients for *Faecalibacterium prausnitzii, Anaerofustis stercorihominis, Clostridium leptum, Bacteroides ovatus*, *Bacteroides sp. 4_3*, and *Bacteroides sp. 3_1* were −0.875, −0.851, 0.784, 0.8, 0.788, and 0.817, respectively.

By broad comparison of the metagenomes and metaproteomes, CD clustered separately from healthy (Figures 1 and S2), as also seen by prior analysis of 16S rRNA gene sequence data [15] and metabolite data [16] from the same cohort. The distinct clustering according to disease phenotype observed in the metaproteome data was statistically significant (p = 0.004) (Figure 1). The clustering of samples from discordant twin pairs into their respective disease category, instead of with their co-twin, suggests that the disease phenotype was a stronger discriminator than genetics (Figure S2 in Supporting Information S1). Therefore, for the rest of the analyses we only considered disease phenotype for comparisons, not twin status, and the four healthy individuals and six ICD individuals were treated as separate phenotypic groups.

Although healthy and CCD metaproteomes could be distinguished from another, they clustered more closely together compared to the ICD metaproteomes that were clearly distinct (Figure 1 and S2). This also substantiates previous findings that there is a more substantial dysbiosis of the gut microbiota associated with ICD [11], [13], [15]. Therefore, we primarily focused on functions that differentiated ICD from healthy, but included comparisons to CCD when relevant.

### Taxonomic profile differences

**Figure 2:**
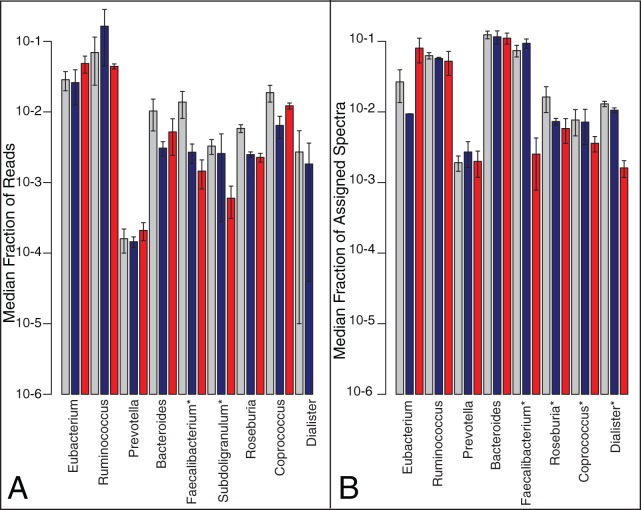
Taxonomic assignments in metagenome and metaproteome datasets. Relative abundance (log scale) of genera in (**A**) metagenomic datasets, determined by reference genome alignments and (**B**) metaproteomic datasets, determined by HMRG PSMs. Error bars represent standard error of the mean of the samples from Healthy (3 MG, 4 MP), ICD (5 MG, 6 MP) and CCD (2 MG/MP). Asterisks indicate genera that were statistically lower in relative abundance in ICD compared to Healthy (q-values of 0.0030, 0.0041, 0.0041, 0.0040 for *Faecalibacterium Roseburia, Coprococcus* and *Dialaster*, respectively). *Subdolidogranulum* was not included in the HMRG database, so it is not shown in the metaproteome. Grey bars  =  Healthy, Blue bars  =  CCD, Red bars  =  ICD. standard error of the mean.

Taxonomic profiles of the metagenomic data were determined using nucleotide alignments and compared based on disease status (healthy, CCD, ICD). Greater than 60% of the metagenomic sequence reads in the samples from healthy subjects could not be assigned at the phylum, family or genus level, compared to ∼40% of the reads in ICD or CCD subjects, potentially reflecting the reduced bacterial diversity in the gut of CD patients. Of the metagenomic reads for which a taxonomic assignment could be made, 396 genera were represented in all of the samples, and nine of those were present at >5% of reads, representing the core taxa. Some members of the Firmicutes phylum, such as *Faecalibacterium*, were significantly depleted in ICD compared to healthy (p<0.05; Figure 2A), a result consistent with 16S rRNA gene sequencing gene sequencing of the same samples [15].

In the metaproteome data we also found a sigificant depletion of proteins from members of the Firmicutes phylum in ICD, p = 0.00025 (Figure 2B). For example, proteins from *Faecalibacterium*, *Roseburia, Dialister* and *Coprococcus* were significantly less abundant in ICD relative to healthy subjects (Figure 2B; Table S8). This finding demonstrates that the systems biology approach used was consistent at both the gene and protein level.

### Broad metagenome-metaproteome comparisons

**Figure 3:**
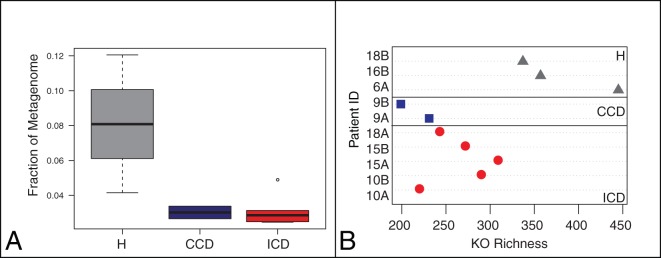
Comparison of protein expression levels across disease categories. (**A**) Boxplots depicting the distribution of the fraction of the metagenomes with PSMs. Boxes indicate 25^th^, 50^th^ and 75^th^ percentile, with whiskers representing 10^th^ and 90^th^ percentile points. (**B**) Gene family richness as measured by the number of KEGG Orthologous group (KO) matches in the metagenomic dataset. Grey  =  Healthy, Blue  =  CCD, Red  =  ICD.

A larger proportion of genes in the metagenomes were expressed and identified as proteins in healthy subjects compared to CD patients (8% H versus 2% ICD or 2% CCD) (Figure 3A). This finding was also supported by a significant decrease in functional richness in the metagenomes of individuals with CD, examined comparing KEGG Orthologous groups (KOs) identified in each sample (Figure 3B). Due to the redundancy of orthologous genes in the HMRG and MM databases, microbial ORFs, which shared >80% sequence identity were clustered into orthologous clusters (OCs), reducing 890,000 ORFs to 68,000 clusters. This generated a total of 5,692 and 3,101 orthologous clusters (OC) from the HMRGs and MMs, respectively, across all metaproteome datasets. Of the OCs that were identified using the MM searches, 344 were identified across all subjects (core) and included general housekeeping proteins (such as ribosomal proteins); whereas 1,221, 720, and 145 OCs were unique to either the healthy, ICD, or CCD core metaproteomes, respectively (Table S9). Analysis of these OCs revealed that 1,017 proteins from the MM searches were unique (i.e., they were singletons), in contrast to all identified proteins from the HMRG search, suggesting that there is considerable protein diversity within the human gut microbiota that is not captured in current reference genome sequences.

Each dataset contained a subset of genes and proteins of unknown function. For example, ∼17% of predicted ORFs in the metagenomic data were either conserved with no known function or were not homologous to any known proteins. Approximately 31% of the proteins identified with the HMRG database (Table S6) and 29% of proteins identified using MM microbial OCs (including proteins that did not cluster) had no known functions (Table S6). Interestingly, one OC comprising 11 unknown proteins was significantly correlated with ICD, whereas five OCs (10–100 s of unknown proteins) were significantly correlated with healthy subjects. These findings support the need for better coupling of phenotypic assays with -omics strategies to aid in the characterization of potentially important unknown genes and proteins.

### Differences between ICD and healthy metaproteomes

**Figure 4:**
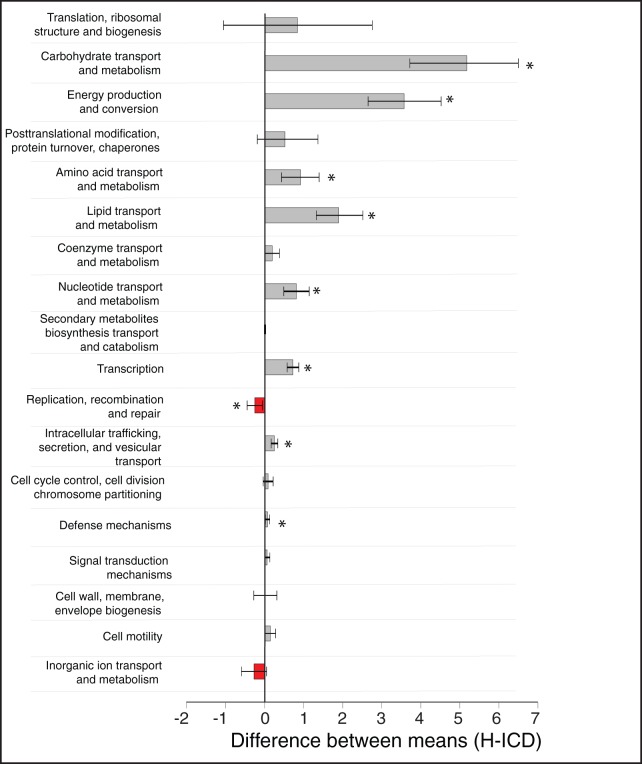
Metaproteome differences between mean Healthy and mean ICD COG frequencies. To determine statistically significant differences between categories, White's non-parametric t-test was used with bootstrapping and Storey FDR multiple test correction. 95% upper and lower confidence intervals are shown. Red and grey bars indicate COG categories that are higher in ICD or Healthy metaproteomes, respectively; Asterisks indicate COG categories that were significantly different between ICD and healthy (q-value<0.05).

There were significant differences in several COG categories when comparing the metaproteomes of ICD to healthy, primarily due to a decrease in abundance of proteins in ICD (Figure 4). General COG categories that were significantly less represented in ICD compared to healthy included “carbohydrate transport and metabolism”, “energy production and conversion”, “amino acid transport and metabolism”, “lipid transport and metabolism”, “nucleotide transport and metabolism”, “transcription, “intracellular trafficking”, and “defense mechanisms”; suggesting that these general processes are deficient in ICD (Figure 4). Only one category, “replication, recombination and repair”, was significantly higher in the ICD metaproteomes compared to healthy (Figure 4).

At a finer scale of resolution, there were 116 statistically significant differentiating specific COGs between disease categories in the metaproteome data (spectra count difference ≥5 and adjusted p-value (q-value) of ≤0.05; Table S10 for complete listing). In particular there was a depletion of microbial proteins in ICD compared to healthy. The general depletion of microbial proteins in ICD could either result from decreased expression, increased protein degradation, or decreased microbial diversity (i.e. reduction of Firmicutes). However, nine COGs belonging to “translation”, “carbohydrate metabolism”, “amino acid metabolism” and “inorganic ion metabolism” (i.e., COG 4771, an outer membrane receptor for ferrienterochelin and colicins), were statistically more abundant in ICD relative to healthy metaproteomes, suggesting that they are potential stool indicators of ICD.

### Metabolic pathways that differentiate ICD and healthy phenotypes

**Figure 5:**
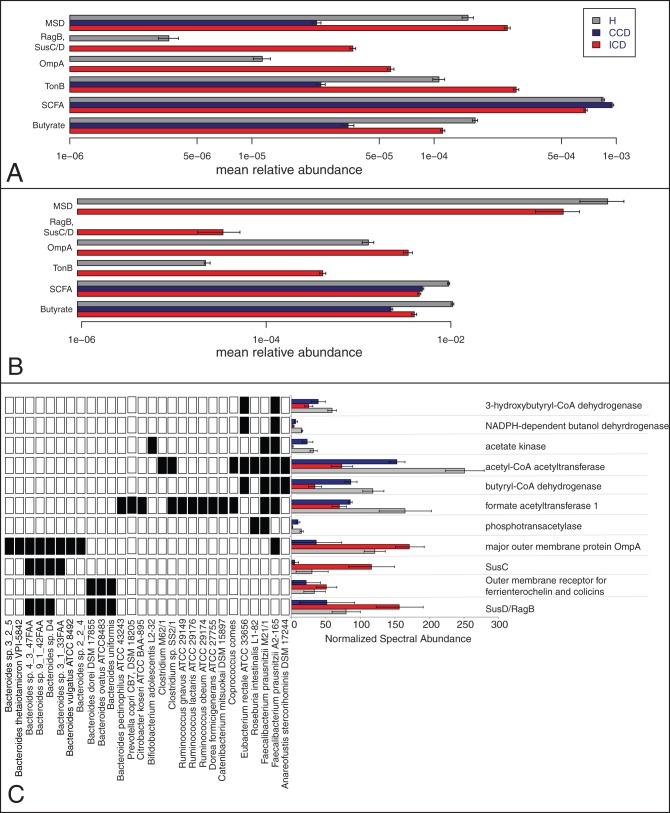
Specific genes and proteins that differ in relative amounts according to disease state. Relative Abundance of mucin-desulfating sulfatase (Mds), RagB and SusC/D, Outer Membrane Protein A (OmpA), TonB, Short-Chain Fatty Acid production (SCFA) and Butyrate production in (**A**) metagenomes and (**B**) MM metaproteomes. Error bars in (A) and (B) represent the standard error of the mean of the samples from Healthy (3 MG, 4 MP), ICD (5 MG, 6 MP) and CCD (2 MG/MP). (**C**) Specific outer membrane proteins and proteins involved in SCFA pathway that differed between disease categories. Protein abundances were calculated as normalized spectral abundance using the HMRG database search. The presence-absence heatmap indicates which of the 51 bacterial strains each protein matched to in the HMRG database search: black  =  species present, white  =  species absent. Grey  =  Healthy, Blue  =  CCD, Red  =  ICD.

The metaproteome data indicated significant differences in carbohydrate degradation pathways between ICD and healthy (Figure 4). Similar to a recent study [24] we also found by screening the metagenomes that the healthy subjects had a higher abundance of genes encoding carbohydrate active enzymes “CAZymes” typical of those that degrade complex carbohydrates in the plant cell wall (e.g. glycoside hydrolases: GH78, GH9, GH30, GH28 and GH26 and polysaccharide lyase PL11), compared to those for degradation of animal-type carbohydrates such as starch and glycogen (e.g. glycoside hydrolases: GH33, GH0109, GH92 and GH89) (Figure S3 in Supporting Information S1). By contrast, the ICD subjects had lower relative amounts of genes encoding CAZymes for degradation of both plant and animal-type carbohydrates compared to healthy. Because IBD and Crohn's patients, in particular, are discouraged from eating fibrous foods, these changes could reflect functional shifts driving these dietary recommendations. However, we do not have detailed metadata about the diet of these subjects. Additionally, the abundance of the protein in CAZy family GH112, which is involved in mucin degradation [25], was depleted in ICD compared to healthy (p<0.01) (Figure 5B), despite more of the corresponding genes (i.e. mucin-desulfating sulfatase (Mds) genes) in ICD (Figure 5A). Mucin desulfation is a rate-limiting step in mucin degradation by colon bacteria [26]. In the colon, secreted mucins have oligoscaccharide side chains that are more heavily sulfated than the side chains of secreted mucins in regions of the digestive tract with lower bacterial numbers. Sulfation of mucins could make them less susceptible to degradation by bacterial glycosidases.

**Figure 6:**
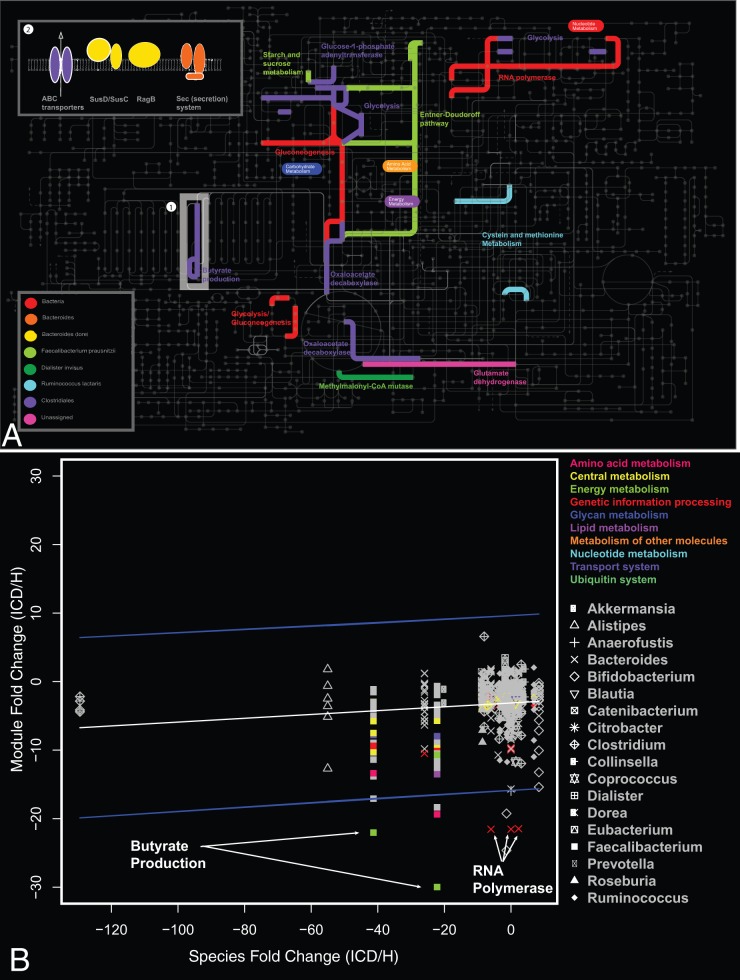
Metabolic Pathways that Differentiate Healthy and ICD phenotypes. (**A**) Metabolic pathways differentiating between healthy and ICD according to metabolic module analysis (p<0.05; 5% FDR). All pathways are less abundant in ICD compared to healthy except for *Bacteroides* membrane proteins (upper left box) that are more abundant in ICD. The colors reflect their phylogenetic origin that was determined using the lowest common ancestor of their HMRG mappings. Grey highlighted areas discussed in the main text: (1) butyrate production; (2) membrane proteins. (**B**) Observed metabolic module abundance shift versus its expected value based on the abundance of the host species. To separate out modules whose fold change is higher/lower than expected by the difference in its species abundance, we used the prediction interval of a fitted linear model (blue lines). The grey symbols are (species-separated) modules that are not significantly different between ICD and H (Wilcoxon rank-sum test; 5% FDR). They could have a high median fold change, but this is not always significant (eg when interpersonal variation is high). The colored symbols are (species-separated) modules that are significant between ICD and H (Wilcoxon rank-sum test; 5% FDR). Colored symbols inside the interval are significantly different but are in line with what would be expected from the species difference. Colored symbols outside the blue lines are higher/lower than expected. Specific *Faecalibacterium* proteins that are down regulated in the butyrate module (green squares) include the following: butyryl-CoA dehydrogenase (EC 1.3.99.2), 3-hydroxyacyl-CoA dehydrogenase (EC 1.1.1.35), enoyl-CoA hydratase/carnithine racemase, and acetyl-CoA acetyltransferases; as well as the module for lysine fermentation to acetate and butyrate (pink square). Specific *Bacteroides* proteins that are down regulated in the DNA-directed RNA polymerase module are the following (red X's): alpha and beta subunits (EC 2.7.7.6).

There was also a depletion of butyrate and other short-chain fatty acid (SCFA) production pathways in ICD in both the metagenome (Figure 5A) and metaproteome (Figure 5B) datasets; corresponding to a depletion of members of the Firmicutes (Figure 5C). KEGG pathway analysis of the metaproteomic datasets also revealed that central metabolic pathways, such as glycolysis, were under-represented in ICD compared to healthy (Figure 6A). Butyrate is known to be a major energy source for colonocytes, is involved in the maintenance of colonic mucosal health and can elicit anti-inflammatory effects, thus its depletion could be one reason for the inflammation in CD. In addition, the reduction of proteins involved in butyrate production in *Faecalibacterium* was even lower than would be expected by the abundance of this organism (Figure 6B), suggesting that their expression was down regulated.

### Bacterial-host interactions and defense

Some specific genes and proteins had a higher relative abundance in ICD. For example, by close examination of both gene and protein abundance measurements we found that several Gram-negative bacterial outer membrane proteins (e.g. OmpA, RagB, SusC/D and TonB) had a higher representation in the ICD microbiota compared to healthy (Figure 5). Based on matches to the HRMG database, these proteins largely corresponded to *Bacteroides* proteins (Figures 5C and 6A). These different membrane proteins have different predicted roles. For example, TonB-dependent receptors take up large macromolecular complexes, including iron/siderophore complexes, vitamin B12 and sulfate esters [27]. OmpA, a pore-forming protein in the outer membrane of many Gram-negative bacteria, harbors diverse functions including maintenance of cell structure, binding various substances, adhesion, and resistance to antimicrobials [28], and is suggested to be involved in gut mucosal association [29]. One hypothesis is that because OmpA is highly represented and highly conserved in many enteric bacteria, the immune system has acquired the ability to recognize and to be activated by this class of protein [30]. Because these proteins are more abundant in ICD, the immune system may respond with a heightened immune response. Our study also provides the first evidence of elevated abundance of other major OMPs, such as RagB, SusC/D associated with CD (Figures 5 and 6A). An elevated IgG response to RagB was previously reported in subgingival samples of patients with periodontitis [31] and virulence of the *rag* locus was demonstrated in *Porphyromonas gingivalis* strains [32]. While the role of RagB/Sus in the etiology of CD warrants further study, our data suggest that there is a shift from a healthy microbiota towards a microbial consortium that can elicit an inflammatory immune response. This finding would support the current hypothesis that CD is manifested by an aberrant mucosal response to otherwise harmless bacterial antigens in genetically susceptible individuals [33], [34]. These differences could also be due to broad shifts in Gram-negative versus Gram-positive bacteria, since we see a reduction in Gram-positive Firmicutes relative to Gram-negative Proteobacteria based on 16S studies [11], [12], [15]. Although there was no observed shift in total *Bacteroides*, previously we found that there were differences in proportions of specific *Bacteroides* species in individuals with ICD compared to healthy [11].

### Broad functional comparisons of the human proteome

Because we are able to measure *both* bacterial and human proteins in the same samples using metaproteomics, a total of 1,646 human proteins were experimentally identified in addition to the microbial proteins discussed above. Gene ontology (GO) analysis revealed that human proteins found in all 3 subject groups (core) are enriched in functions associated with the structural integrity of the mucosal epithelium such as regulation and activity of actin cytoskeletal components. Proteolysis, digestion, and carbohydrate catabolism were also among the most abundant ‘core’ functional terms, as would be expected in the human GI-tract (Figure S4A in Supporting Information S1). For human proteins that varied in healthy compared to CD, the majority were involved in epithelial integrity and function, as detailed below.

### Impaired epithelial integrity in ICD

The observation of several human proteins detected in higher abundance in CD supports the hypothesis that subjects with ICD, even in remission, have a defective epithelial barrier. The higher abundance of human proteins could also be a consequence of surgical resection of the ileum. For example, a higher abundance of proteins involved in inflammatory and host defense, wounding response, intracellular transport, and epithelial development and differentiation were enriched in ICD subjects (Figure S4B in Supporting Information S1). Furthermore, other proteins that function in maintaining mucosal integrity were identified as being statistically under-represented in ICD (q-value = 0.022), including protocadherin LKC, a calcium dependent mediator of cell-cell adhesion that associates with the mucosal actin cytoskeleton [35] and type 1 collagen (alpha-2), the major collagen in the intestinal extracellular matrix [36]. A depletion of these proteins might compromise host defense at the mucosal interface.

A defective epithelial barrier is thought to result in an aberrant host response to luminal antigens leading to an exaggerated adaptive immune response and chronic inflammation [37]. Human alpha defensin 5, a protein implicated in regulation of bacterial concentrations in the ileal intestinal crypt [38]–[40] was also statistically more abundant in ICD (q-value = 0.022), suggesting that the host may increase expression of defensins in response to aberrant microbiota in these subjects, or that the products are leaking from the intestinal site of action and therefore detected in higher amounts in the stool samples.

### Impaired intestinal absorption in ICD

Several pancreatic enzymes that are largely broken down in the small intestine: chymotrypsinogen B1 and B2, pancreatic carboxypeptidase A1 and B1 and pancreatic lipase, were identified with higher abundance in stool samples of the subjects with ICD. These enzymes are synthesized in the pancreas as inactive precursors that are activated in the intestine where they aid in digestion. Relatively high amounts of pancreatic enzymes in stool samples may be indicative of pancreatitis, which has been linked to CD [41], but remains to be confirmed since the subjects in this study did not have active pancreatitis at the time of sampling.

## Discussion

In this study we used a combination of large and complementary “-omics” datasets to provide the most comprehensive view of the functional role of the gut microbiota in CD to date. We studied the same stool samples obtained from twelve individuals that were previously characterized with respect to microbial community and metabolite compositions as part of a large CD twin cohort [11], [12], [15], [16]. Here our aim was to specifically gain insight into functional differences at the gene and protein level that were correlated to Crohn's disease. The results of this study not only support existing lines of evidence but also add more pieces of information to help fill in the complex puzzle of CD etiology. Similar to the previous studies of 16S rRNA genes [11], [12], [15] and metabolites [16], this study also found that the proteins extracted from the samples clustered separately according to disease status. Together these different omics datasets provide an enormous amount of information, with dozens of species, thousands of metabolites and hundreds of proteins that vary in relative amounts, particularly when comparing ICD to healthy. The majority of the metabolites [16] and many of the proteins that differed according to disease status have not yet been characterized and their functions are unknown. Specifically, the unknown proteins detected here that were expressed in higher amounts in ICD are of particular interest for further exploration because they were expressed and not merely hypothetical proteins predicted from sequence data and therefore potentially play functional roles of importance to ICD.

The value of the eco-systems biology approach used here comes from the ability not only to examine the structure and function of the microbiota from multiple perspectives, but also from the ability to integrate data from the gut microbiota and the host. New findings from this study suggest several malfunctions in ICD, both with respect to the intestinal microbiota and the host. For example, dysbiosis of the bacterial community in ICD resulted in expression of higher levels of several bacterial cell surface proteins, many of which are antigenic and could contribute to an exaggerated immune response. This imbalance came at the expense of loss of proteins produced by many beneficial members of the microbiota, including proteins involved in butyrate production and degradation of mucin, thus supporting the previously observed decrease in abundance of the corresponding species in the same samples using 16S rRNA gene fingerprinting approaches [11], [12]. At the same time, there were several preliminary indications that the host epithelial barrier was impaired, both with respect to structural integrity of the mucosal boundary and with respect to its ability to absorb secreted enzymes; although these findings could also be a consequence of ileal resection. This finding correlates to the previously reported increase in bile acid metabolites in the same samples from the ICD individuals [16].

Together these large omics datasets point towards several new targets for further investigation in the pursuit for diagnosis and therapeutic treatments for Crohns disease. This study also highlights the value of using an eco-systems biology approach to obtain a more complete picture of the complex interactions between the thousands of bacterial species in the distal gut with the human host. It will be of great value to extend these studies to larger cohorts of CD patients and to carry out longitudinal studies to assess i) how the composition and function of the gut microbiota changes over time with respect to disease inflammation and ii) how the microbiota is impacted by other factors including drug therapy and surgery.

## Materials and Methods

### Patient cohort

The Swedish twin cohort was previously described in several studies [11], [12], [15], [16], [42], [43]. For this study, we focused on six monozygotic twin pairs including: one set of healthy twins with existing metaproteome data [18] one set of concordant twins with Crohn's disease inflammation localized in the colon (CCD), two sets of concordant twins with Crohn's disease inflammation localized in the ileum (ICD) and two sets of ICD discordant twins (Table S1 in Supporting Information S1). Representatives of both sexes were included in the study (6 females and 6 males) and the subjects were all adults (youngest, born 1962; oldest born 1947). None had taken antibiotics within 12 months of sampling. Three of the subjects had gastroenteritis within 3 months prior to sampling. Most of the patients had undergone surgery as indicated, but all were many years prior to the sampling event (Table S1 in in Supporting Information S1). All patients were in endoscopic remission, or had minor inflammatory activity in the neo-terminal ileum only, at the time of sampling. In addition, the 16S rRNA gene composition was determined for all samples previously by 454 pyrotag sequencing [15] and the metabolite compositions were determined from fecal water collected from the same samples [16].

### Community DNA preparation

Stool samples were shipped to the Orebro University Hospital, Orebro, Sweden, at most one day after sample collection and immediately frozen at −70°C upon arrival. The samples were stored continuously frozen until use and small portions were excised and thawed immediately prior to DNA extraction to avoid freeze-thaw damage. DNA was extracted from 250 mg of each stool sample in duplicate using the MoBio Power Soil DNA Kit (MoBio, Solana Beach, CA, USA), as previously described [15], and if necessary to get higher yields we also used an optimized IGS-Zymo DNA extraction protocol reported previously [44].

### Shotgun metagenomic sequencing

DNA isolation from stool samples yielded 3–5 ug of purified metagenomic DNA from each of twelve samples. Each sample was subjected to picogreen and gel-based QC assays prior to library construction. Unpaired, shotgun fragment sequencing libraries were constructed using our customized, automated library construction procedure. Our method modifies the manufacturer-provided protocol by adjusting enzymatic reaction volumes and replacing gel-based fragment size-selection steps with AMPure SPRI magnetic beads to enable automation of the process using liquid-handling robotics. Following library construction, each sample was subjected to emPCR amplification and 454 sequencing according to manufacturer specifications. Raw sequence data was processed using the Roche/454 run processing software to filter short, mixed, and low-quality reads. Whole metagenomic shotgun sequencing generated a total of 15,307,850 reads and more than 5,428,202 kilobases (or 5 Gbp) of high-quality, passed-filter sequence data (Table S2 in Supporting Information S1).

The metagenome sequence data can be retrieved using the following URL for the NCBI SRA data deposit, under project ID 46321: http://www.ncbi.nlm.nih.gov/sites/entrez?db=bioproject&cmd=Retrieve&dopt=Overview&list_uids=46321.

### Metagenomic taxonomic classification

Metagenomic reads were compared to publically available human-associated bacterial reference genomes using NUCMER (80% id, 80% coverage) for taxonomic assignment. In cases where reads did not match reference genomes taxonomic classification was made using sequence comparison against known proteins in NCBI NR using BLASTX (90% id). In cases where reads had high identity matches to multiple sequences, the taxonomic nearest neighbor was chosen. Taxonomic classification for each MS spectrum was determined by the protein sequence predicted from metagenomic contig sequences, where the taxonomy of a contig is based on the nearest neighbor classification of the read sequences composing the contig. In cases where no classification was obtained, the ‘human gut microbiome classification’ was given. Family assignments are based on the NCBI taxonomic tree. Potential 16S sequences were identified using RNA-HMM and classified using RDP 2.0. Clustering of samples by taxonomy was done using Ginko, with a log10(X+1) normalization, euclidean distances and Ward's method for hierarchical clustering.

### Metagenomics gene finding and protein clustering

Sequences were assembled with the Newbler Assembler (v2.0.01.14) and genes were predicted on contigs greater than 500 bp using METAGENE [45]. Genes on contigs less than 500 bp were searched against a database of reference genomes using FASTX [46]. Genes were predicted from alignments to homologous sequences. In regions where no homologous sequences are found, METAGENE [47] was used for de novo gene prediction and generated 594,362 genes, greater than 50 nt, across 10 metagenomic datasets.

An all-vs-all BLASTP [47] search was performed against the human associated bacterial reference genome protein database using thresholds of percent identity >80 and e-value <10−5, protein clusters were created using an MCL [48] with an inflation value of 1.5. Predicted ORFs from metagenomes were mapped to 17,408 of these clusters using BLASTP with an 80% identity threshold; 196,002 genes did not map to a cluster.

### Functional analysis

ORFs were searched against the eggNOG [49], CAZY [50] and KEGG Orthologous groups [51] databases using NCBI-BLAST [47] using e-value cutoff of 10^−6^ and bits per position cutoff of 1. COG and NOG functional assignments were assigned based on this comparison. In addition sequences were searched against a library of HMMs consisting of TIGRFAMS [52], and PFAM [53], [54] using HMMPFAM [55]. Relative abundances of annotations were determined using a random sampling of the smallest number of reads in contigs as the sample size with 100 iterations. The mean of this random sampling was calculated to determine the relative abundance of a gene or function in the sample.

### Cell lysis and protein extraction

Approximately 10 g portions of the same stool samples used for DNA extractions were processed by differential centrifugation to enrich the bacterial cell fraction as previously described [18]. The microbial cell pellets (∼100 mg) were processed via single tube cell lysis [56] protein digestion and peptide desalting prior to 2d-LC-MS/MS analyses [18], [57]. Briefly, the cell pellet was resuspended in 6 M Guanidine/10 mM DTT to lyse cells, denature proteins, and reduce disulfide bonds. The guanidine concentration was diluted to 1 M with 50 mM Tris buffer/10 mM CaCl2 and sequencing grade trypsin (Promega, Madison, WI) was added to digest proteins to peptides. Following proteome digestion, the peptide solution was treated again with 10 mM DTT to reduce disulfide bonds. We have found this method of double reduction to be as effective as blocking with iodoacetamide. The complex peptide solution was desalted via C_18_ solid phase extraction, concentrated, solvent exchanged into 100% water/0.1% formic acid, filtered (0.45 um filter), and aliquoted.

### 2D-LC-MS/MS

All samples were analyzed in technical duplicates via two-dimensional (2D) nano-LC MS/MS with a split-phase column (RP-SCX-RP) [58], [59] on a LTQ Orbitrap (Thermo Fisher Scientific) with 22 hr runs per sample. For each sample, peptide mixtures were separated by a 12 step, multidimensional high-pressure liquid chromatographic elution consisting of eleven salt pulses (ammonium acetate) followed by a 2 hr reverse-phase gradient from 100% solvent A (A: 95% H_2_O, 5% acetonitrile, 0.1% formic acid) to 50% solvent B (B: 30% H_2_O, 70% acetonitrile, 0.1% formic acid). The last salt pulse was followed with a gradient from 100% solvent A to 100% solvent B. During a single chromatographic separation (22 hr run), mass spectral data acquisition was performed with Xcalibur software (version 2.0.7; Thermo Fisher Scientific). Precursor full MS spectra (from 400–1700 m/z) were acquired in the Orbitrap with resolution r = 30,000 followed by five data-dependent MS/MS scans at 35% normalized collision energy in the LTQ with dynamic exclusion enabled (repeat count 1).

### Protein database construction

The first database, referred to as the matched metagenome (MM), was created per sample by directly predicting ORFs from raw sequencing reads to prevent loss of sequence diversity when collapsing unrelated sequencing reads for metgenome assembly (RMPS metagenomic processing method described in detail by Cantarel et al. [23]. ORFs larger than 50 nt were predicted using Metagene. Redundant protein sequences were removed, by pairwise comparisons using 100% identity over 100% of the shorter proteins (i.e. when aligning 2 proteins, the shorter of the two must be covered completely by the larger one at 100% identity), producing 491K – 1.58 M ORFs per sample. Each of these 12 individual protein databases (6a, 6b, 9a, 9b, 10a, 10b, 15a, 15b, 16a, 16b, 18a, and 18b) included human reference sequences (July 2007 release, NCBI; ∼36,000 protein sequences) and common contaminants (i.e., trypsin and keratin; 36 protein sequences).

A second database, referred to as the human microbial isolate reference genome database (HMRGs), was utilized in a complementary database search and also contained human reference sequences and common contaminants. While this reference genome database is not exactly representative of each sample, it can provide definitive species/protein identifications, which were used to support and complement the MM searches. This database was created by concatenating 51 human-derived reference isolate genomes from the JGI IMG human microbiome project (IMG-HMP) into a single FASTA-formatted protein sequence database. The criteria used to select 51 human-derived microbial isolates were based on genera that have been previously found in the 16S data from the same samples [15] in addition to strains that are known to be common gut inhabitants; while avoiding representation from similar species and strains to reduce redundancy. A list of all 51 isolates included in this database can be found in Table S7. All protein databases, MM and HMRG datasets, and supporting figures and tables can also be downloaded from: http://compbio.ornl.gov/crohns_disease_metagenomics_metaproteomics/.

### Proteome informatics

All MS/MS from individual runs were searched with the SEQUEST (v.27) algorithm [60] against a custom-made FASTA formatted protein sequence databases described below. The SEQUEST database search required fully tryptic (tryptic at both termini) peptides with up to 4 miscleavages and a 3 Da mass tolerance window around the precursor ion mass and 0.5 Da for fragment ion masses. As previously described [23], all SEQUEST output files were assembled and filtered using DTASelect (v1.9) [61] at ≥2 peptides per protein for the HMRG database searches and at a 1-peptide level (required minimum of ≥1 peptides to confidently identify theoretical peptides from a genomic read followed by ≥2 peptides to identify a protein) for the MM database searches with the following widely accepted parameters: cross correlation scores (XCorr) of at least 1.8, 2.5, 3.5 for +1, +2, and +3 charge states, respectively and a minimum deltCN of 0.0 for all 12 samples (24 MS runs). A “post-database” search filter was applied to the MM identifications where we used the high mass accuracy capabilities of the Orbitrap to remove all peptides that did not fall within −10≤ ppm ≤10 to the predicted parent mass of the SEQUEST identified peptide. This was done to remove the large number of false positives generated from the minimum of ≥1 peptides to confidently identify a peptide from a genomic read. This method of filtering peptides via high mass accuracy post-SEQUEST database searches is generally an accepted alternative to filtering during the search via mass accuracy. Both methods have advantages and disadvantages, but for our workflow filtering after the SEQUEST search was found to be most effective.

The acquired mass spectrometry data (mzXML format) from this publication have been submitted to the Proteome Commons Tranche repository at www.proteomecommons.org and assigned the hash identifier: rji3fAXT1XG0PxdrWWrM1M4XXznm6i7XKW2ZMVbfyYvo2G44eBimTcv4osnXHyhDvoCOA1av4EywiTFqX8PfJI9SP4EAAAAAAAChfg.

### False discovery rates

A target-decoy database [62], [63] was generated for the HMRGs and the MMs for one healthy (6b, run 1), ICD (18a, run 2), and CCD (9a, run 2) subject and searched against their corresponding MS experiments (i.e., forward-reverse database for sample 6b was searched against spectra from run 1) to estimate the peptide-level false discovery rate (FDR). All target-decoy SEQUEST output files were assembled and filtered using DTASelect (v1.9) [61] with the same XCorr filters of at least 1.8, 2.5, 3.5 for +1, +2, and +3 charge states, respectively. The HMRGs were filtered at a ≥2 peptide per protein with a deltCN 0.0 with an empirical FDR threshold of ≤2.0%. The MM data was filtered at a ≥1 peptide per predicted genomic read with a deltCN 0.0 and high mass accuracy of parent peptide (−10 ≤ ppm ≤ 10) followed by a post-database ≥2 peptide per protein filter, with an empirical FDR threshold of ≤2.0%. Additional metrics and results on false discovery rates can be found in Supporting Information S1 and Tables S11 and S12.

### Proteome label-free quantification

The spectral count for a microbial protein cluster (“CLST…”) was calculated as the number of unique peptide identifications that can be attributed to proteins from that cluster and not from any other cluster. Because proteins with high sequence similarity were grouped in clusters, the majority of peptide identifications from the metagenomic read databases (RMPS) can be uniquely attributed to only one cluster. The spectral counts for human proteins were calculated from both unique and non-unique peptide identifications using DTASelect with default settings as described above.

Spectral counts for both human proteins and microbial protein clusters from an MS/MS run were normalized by the total numbers of tandem mass spectra (MS/MS) of this run. A scaling factor, α*i*, was calculated for every run as α*i*  =  N/ni, where N is the average number of total MS/MS spectra per run and ni is the MS/MS spectral number of run *i*. The spectral counts for all proteins in a single MS run were then normalized by multiplying them with the run's scaling factor. The reference isolate genome database results were also normalized using the same scaling factor and approach.

The 24 MS runs were grouped into the following three sample sets for both databases (MMs and HMRGs): healthy subjects: 6a, 6b, 16b, and 18b; CCD subjects: 9a and 9b; and ICD subjects: 10a, 10b, 15a, 15b, 16a, and 18a.

### Statistical analyses

The metagenomic microbial protein clusters (MM databases) with differential expression between two sample sets were identified using label-free quantification. We only considered microbial protein clusters that have more than five spectral counts in four or more of the runs in the two sets under comparison. P-values were calculated using the Wilcoxon rank sum test. The p-values were then used to compute q-values [64]. Proteins were considered as differentially expressed if their q-values were less than a false discovery rate threshold of 0.05 and the differences between their median spectral counts of the two sets are greater than 5. Human proteins were quantified separately using the same procedure.

The proteomics results were also analyzed using hierarchical clustering. We only considered proteins with median absolute deviations greater than 1. Normalized spectral counts were log2 transformed by adding a pseudo-count of one. Hierarchical clustering on both proteins and samples were performed using the hclust function in the R stat library and the heatmap was plotted using the heatmap.2 function in the R gplot library.

Non-metric multidimensional scaling (nMDS) was performed using normalized spectral abundances of proteins derived from 24 MS runs searched against 51 human-associated bacterial isolates. nMDS was performed in PCORD v5 using the Bray-Curtis distance measure [65]. Briefly, a matrix of normalized spectral counts for each protein from each metaproteomic run were imported into PCORD v5 and the indicator analysis was performed using the randomization method. MRPP analysis was performed on the rank transformed spectral abundances within PCORD v5 to test the null hypothesis that there is no difference between the bacterial metaproteomic profiles from each phenotype.

KEGG modules analysis was performed to highlight differences in metabolism between healthy and CD. The bulk of metaproteomic KOs were mapped to the KEGG modules reference database in addition to the butyrate production module. Only modules that had more than 30% coverage were considered for downstream analysis. Then differential expression between modules was tested using Wilcoxon's rank-sum test in R and p-values were corrected for multiple testing using Benjamini-Hochberg's false discovery rate (FDR). A module was considered significantly different if the median difference between the two groups was more than 5 with FDR set to 10% under a two-sided alternative hypothesis. Modules and KOs that were significantly down regulated in ICD were visualized within iPATH [66]. Additionally, the phylogenetic origin of these modules and KOs, was shown using the lowest common ancestor.

### Ethics

LBNL has an approved Federal-wide assurance on file with HHS that covers this activity: OHRP Federal-wide Assurance Number FWA 00006253. Certification of Human Subjects Committee review: This activity has been reviewed and approved by the HSC in accordance with requirements sent forth in the DHHS regulations at 45 CFR 46.103(f), which requires that each application or proposal for HHS-supported human subject research be reviewed and approved by the Institutional Review Board. Date of Approval: April 30, 2010; Approval Number: 272H01-30APR11.

The consent procedure was approved by the ethical research committee at Örebro University Hospital, where the samples were collected. The study was approved by Örebro Lans Landsting on December 17, 2003 (D-nr 167/03).

### Consent to participation in the study “Ulcerative colitis and Crohn's disease in twins” and to treatment of personal information

I have been informed in writing about this actual study and have had time in peace and quiet to read through the information and to ask questions by telephone. I have also been provided with a copy of the written information and my written consent.

Through my signature I provide my consent to:

participate in the study.
that my personal information can be used as in the written information.
that my samples are treated as in the written information.
that Jonas Halfvarson, gastroenterologist at USÖ, can request copies of my medical journal.

I am aware that participation is voluntary, and that at the same time I may at any time and without excuse cancel my participation without influencing my future care.

(Direct translation from Swedish).

## Supporting Information

Supporting Information S1 **Additional figures, tables, a note regarding technical and twin reproducibility in the metaproteomes and peptide-level false discovery rates.**
(PDF)

Table S5 **Normalized total spectra counts across all subjects and 24 MS runs for the matched metagenome (MM) database searches.**
(XLS)

Table S6 **Normalized total spectra counts across all subjects and 24 MS runs for the human microbial isolate reference genome database (HMRG) searches.**
(XLS)

Table S7 **Human microbial isolate reference genome database (HMRG) database components.** 51 bacterial isolates were downloaded from the JGI IMG human microbiome project (IMG-HMP) into a single FASTA-formatted protein sequence database.
(XLSX)

Table S8 **Distribution of all normalized ‘unique’ spectra counts (worksheet 1) for a metaproteome genus-level comparison of all 24 MS runs against the HMRG database.** Three comparisons (worksheet 2–4) between different phenotypes (healthy, ICD, and CCD) were performed with Wilson rank sum: Q value (adjusted P value) less than 0.05, difference between medians of the two conditions greater than 5, and more than 4 runs with greater than 5 spectral counts. Only the ‘healthy’ versus ‘ICD’ comparison have several genera that are significantly changed.
(XLSX)

Table S9 **Core and unique microbial protein clusters identified in the metaproteomes.** Common core microbial protein clusters (worksheet 1) identified in the metaproteomes of all subjects included in the study (healthy, ICD and CCD). Microbial protein clusters that were identified as unique to one phenotype, healthy (worksheet 2), ICD (worksheet 3), and CCD (worksheet 4).
(XLSX)

Table S10 **Metaproteomic statistical comparison of spectra assigned to COGs identified across all samples.** Three comparisons (worksheet 2–4) between different phenotypes (Healthy, ICD, and CCD) were performed with Wilson rank sum. A COG labeled with significant UP or DOWN has to satisfy these criteria: Q value (adjusted P value) less than 0.05, difference between medians of the two conditions greater than 5, more than 4 runs with greater than 5 spectral counts.
(XLSX)

Table S11 **False discovery rates estimated at the peptide level (≥2 peptide level) for the HMRG database searches for three MS experiments: 6b, run 1 (worksheet 1), 9a, run 2 (worksheet 2), and 18a, run 2 (worksheet 3).**
(XLS)

Table S12 **False discovery rates of read-based peptides from MM metaproteomic database searches for three MS experiments: 6b, run 1 (worksheet 1), 9a, run 2 (worksheet 2), and 18a, run 2 (worksheet 3) at a 1-peptide level with and without high mass accuracy.** FDRs estimated post-database mapping of all read-based peptides to assembled contigs at a 2-peptide level are provide in the final worksheet.
(XLS)
