# Supplementary material for: Integrated Metagenomics/Metaproteomics Reveals Human Host-Microbiota Signatures of Crohn's Disease
Source: PLoS One. 2012 Nov 28;7(11):e49138. doi: 10.1371/journal.pone.0049138 (PMC3509130; doi:10.1371/journal.pone.0049138)
Supplement: Supporting Information S1 — Additional figures, tables, a note regarding technical and twin reproducibility in the metaproteomes and peptide-level false discovery rates. (PDF) [file pone.0049138.s001.pdf]

Integrated metagenomics/metaproteomics reveals human host-microbiota signatures of Crohn's disease

Supporting Information

Table of Content:

Supporting Figures and Legends

Supporting Tables and Legends

Supporting Note

Supporting Figures

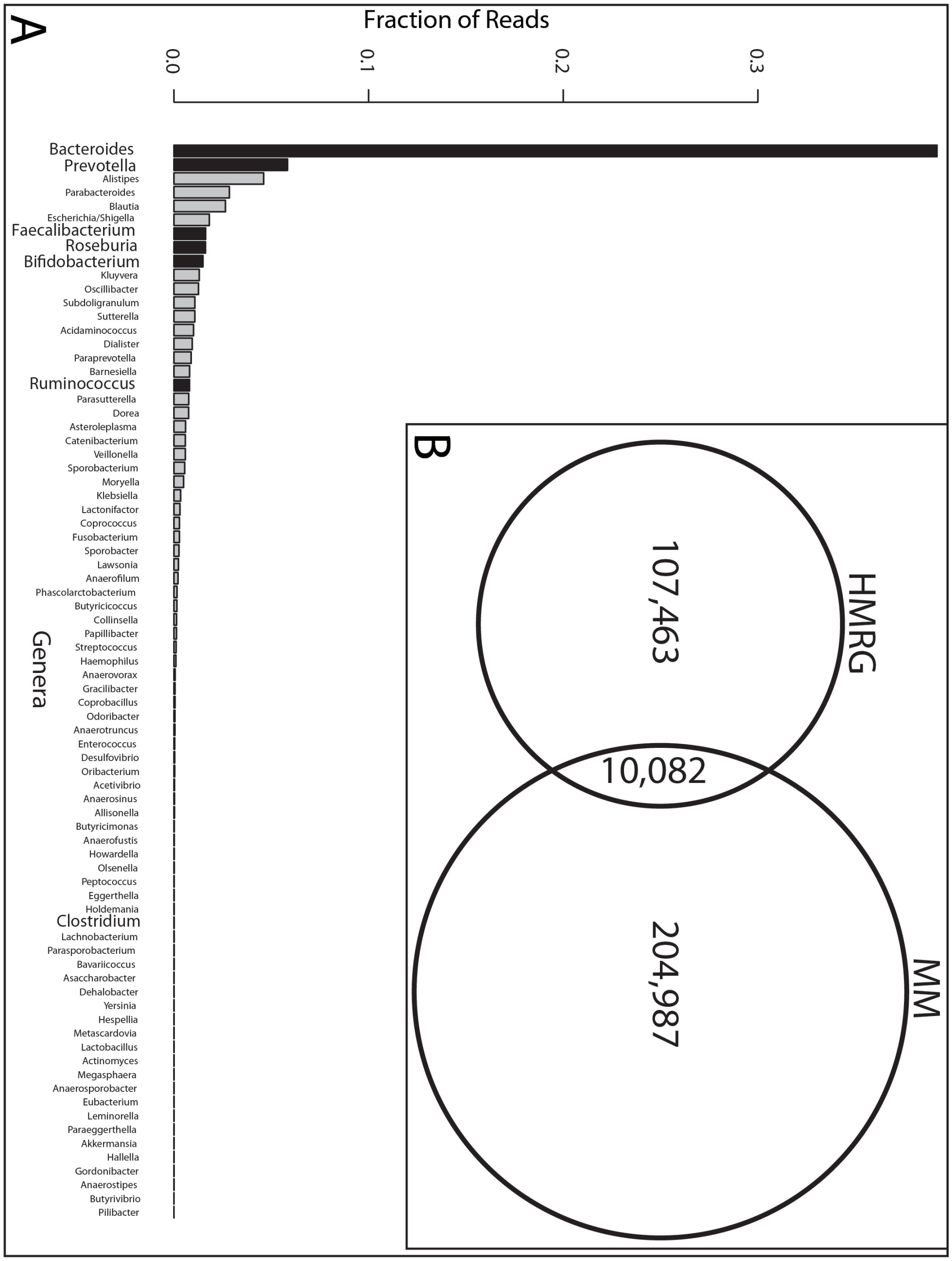

Figure S1. (A) Rank abundance plot of taxa abundances in existing 16S datasets 10 for samples in this study. Genera included in the HMRG database are highlighted in black and a larger font. (B) Venn diagram of assigned non-redundant PSMs from the Matched Metagenome (MM) and Human Reference Genome (HMRG) protein database searches.

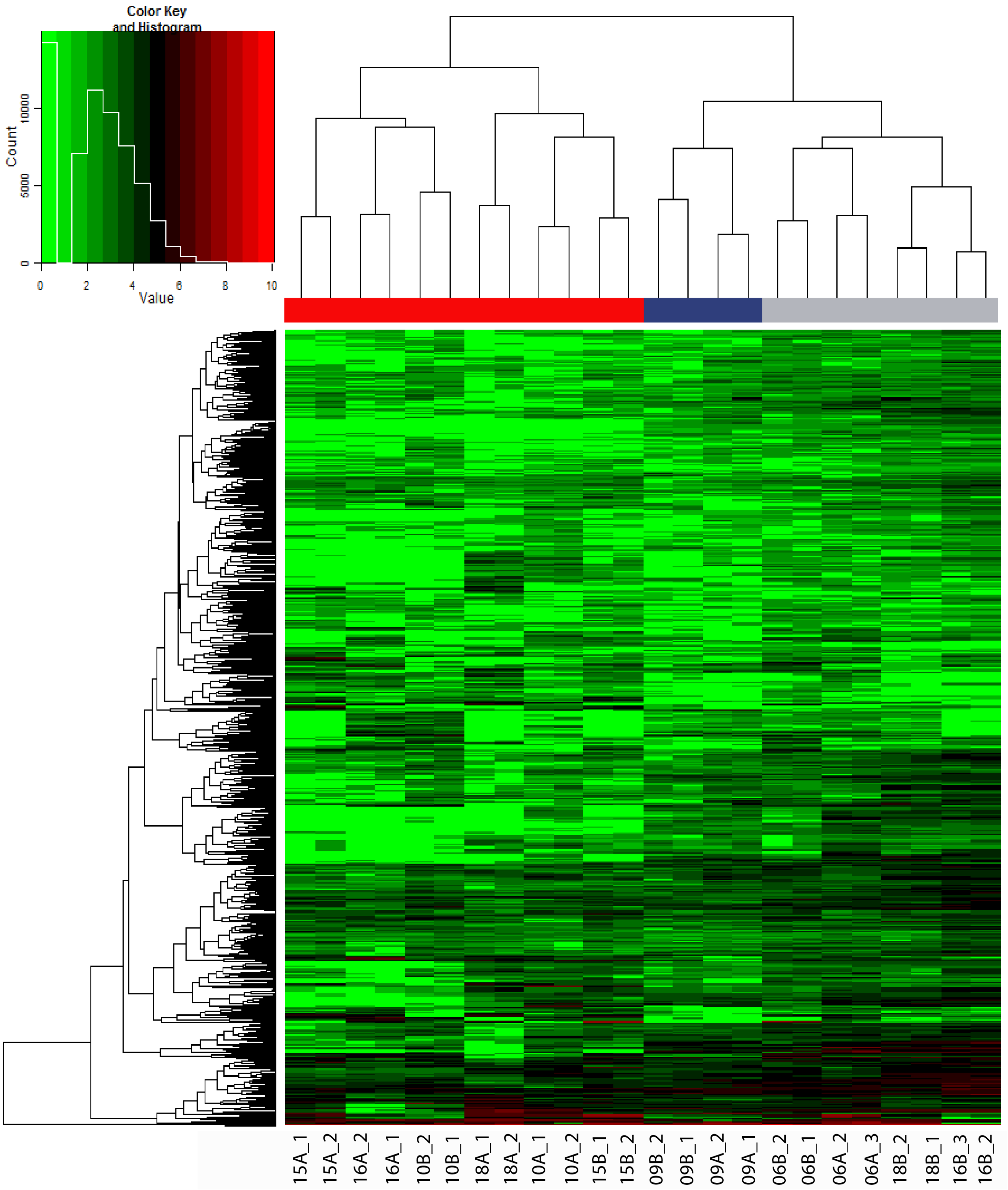

Figure S2. Clustering by Phenotype. Heatmap of metaproteomes predicted from the HMRG database search. Healthy = grey, CCD = blue, ICD = red.

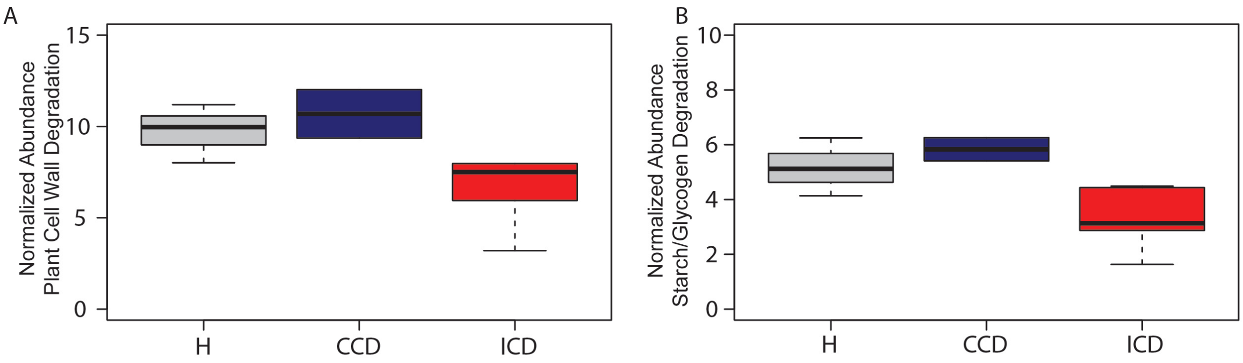

Figure S3. Normalized count of genes involved in (A) plant cell wall carbohydrates degradation and (B) Starch and Glycogen Degradation, based on CAZY enzyme database search

Figure S4. Human proteins identified in the metaproteome data. Bars represent mean protein count of human proteins identified using Gene Ontology (GO) terms found in all individuals (A), and those that are enriched according to disease (B), healthy (green); ICD (red), and CCD (blue).

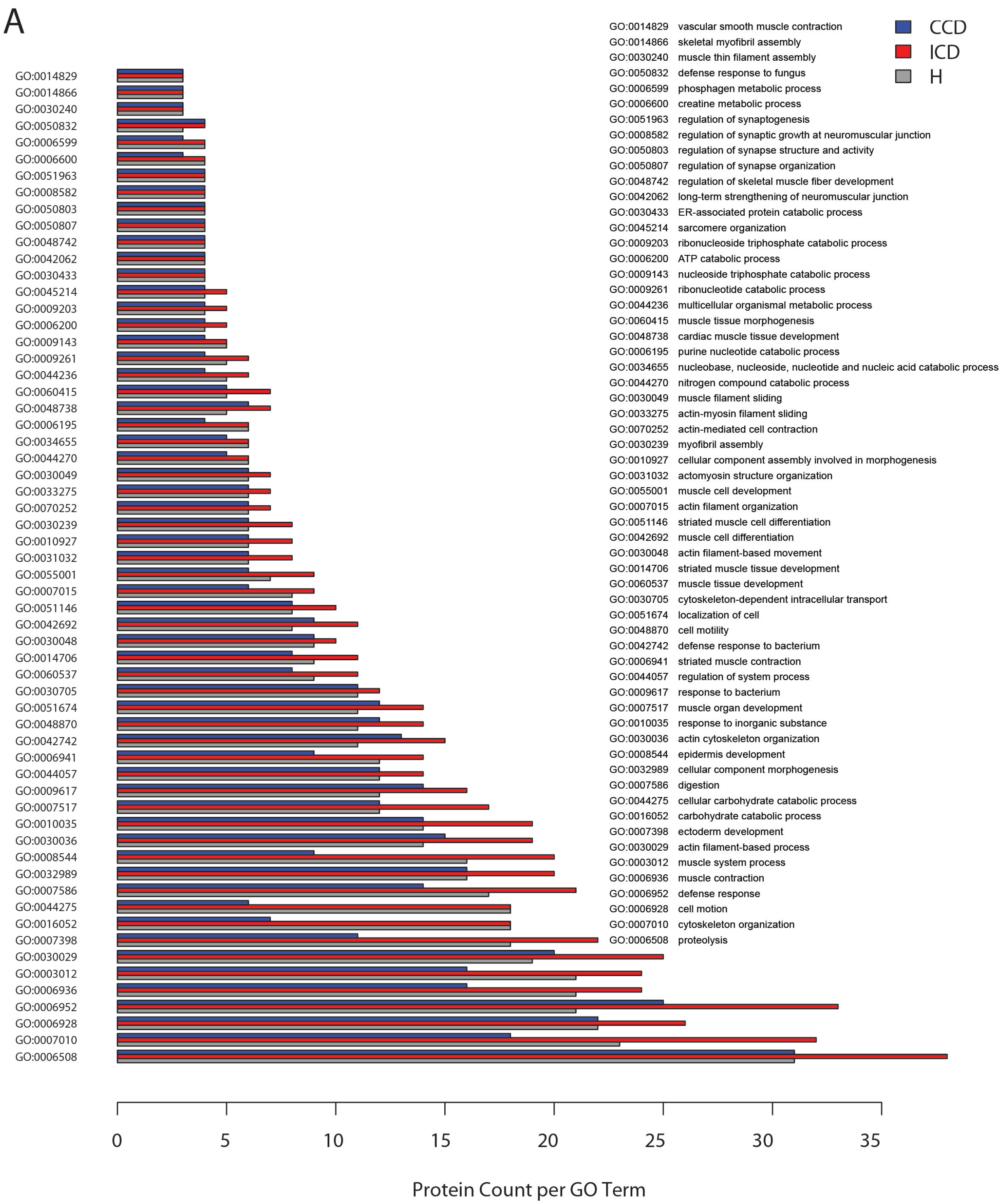
A
CCD
ICD
H
GO:0014829 vascular smooth muscle contraction
GO:0014866 skeletal myofibril assembly
GO:0030240 muscle thin filament assembly
GO:0050832 defense response to fungus
GO:0006599 phosphagen metabolic process
GO:0006600 creatine metabolic process
GO:0051963 regulation of synaptogenesis
GO:0008582 regulation of synaptic growth at neuromuscular junction
GO:0050803 regulation of synapse structure and activity
GO:0050807 regulation of synapse organization
GO:0048742 regulation of skeletal muscle fiber development
GO:0042062 long-term strengthening of neuromuscular junction
GO:0030433 ER-associated protein catabolic process
GO:0045214 sarcomere organization
GO:0009203 ribonucleoside triphosphate catabolic process
GO:0006200 ATP catabolic process
GO:0009143 nucleoside triphosphate catabolic process
GO:0009261 ribonucleotide catabolic process
GO:0044236 multicellular organismal metabolic process
GO:0060415 muscle tissue morphogenesis
GO:0048738 cardiac muscle tissue development
GO:0006195 purine nucleotide catabolic process
GO:0034655 nucleobase, nucleoside, nucleotide and nucleic acid catabolic process
GO:0044270 nitrogen compound catabolic process
GO:0030049 muscle filament sliding
GO:0033275 actin-myosin filament sliding
GO:0070252 actin-mediated cell contraction
GO:0030239 myofibril assembly
GO:0010927 cellular component assembly involved in morphogenesis
GO:0031032 actomyosin structure organization
GO:0055001 muscle cell development
GO:0007015 actin filament organization
GO:0051146 striated muscle cell differentiation
GO:0042692 muscle cell differentiation
GO:0030048 actin filament-based movement
GO:0014706 striated muscle tissue development
GO:0060537 muscle tissue development
GO:0030705 cytoskeleton-dependent intracellular transport
GO:0051674 localization of cell
GO:0048870 cell motility
GO:0042742 defense response to bacterium
GO:0006941 striated muscle contraction
GO:0044057 regulation of system process
GO:0009617 response to bacterium
GO:0007517 muscle organ development
GO:0010035 response to inorganic substance
GO:0030036 actin cytoskeleton organization
GO:0008544 epidermis development
GO:0032989 cellular component morphogenesis
GO:0007586 digestion
GO:0044275 cellular carbohydrate catabolic process
GO:0016052 carbohydrate catabolic process
GO:0007398 ectoderm development
GO:0030029 actin filament-based process
GO:0003012 muscle system process
GO:0006936 muscle contraction
GO:0006952 defense response
GO:0006928 cell motion
GO:0007010 cytoskeleton organization
GO:0006508 proteolysis
0
5
10
15
20
25
30
35
Protein Count per GO Term

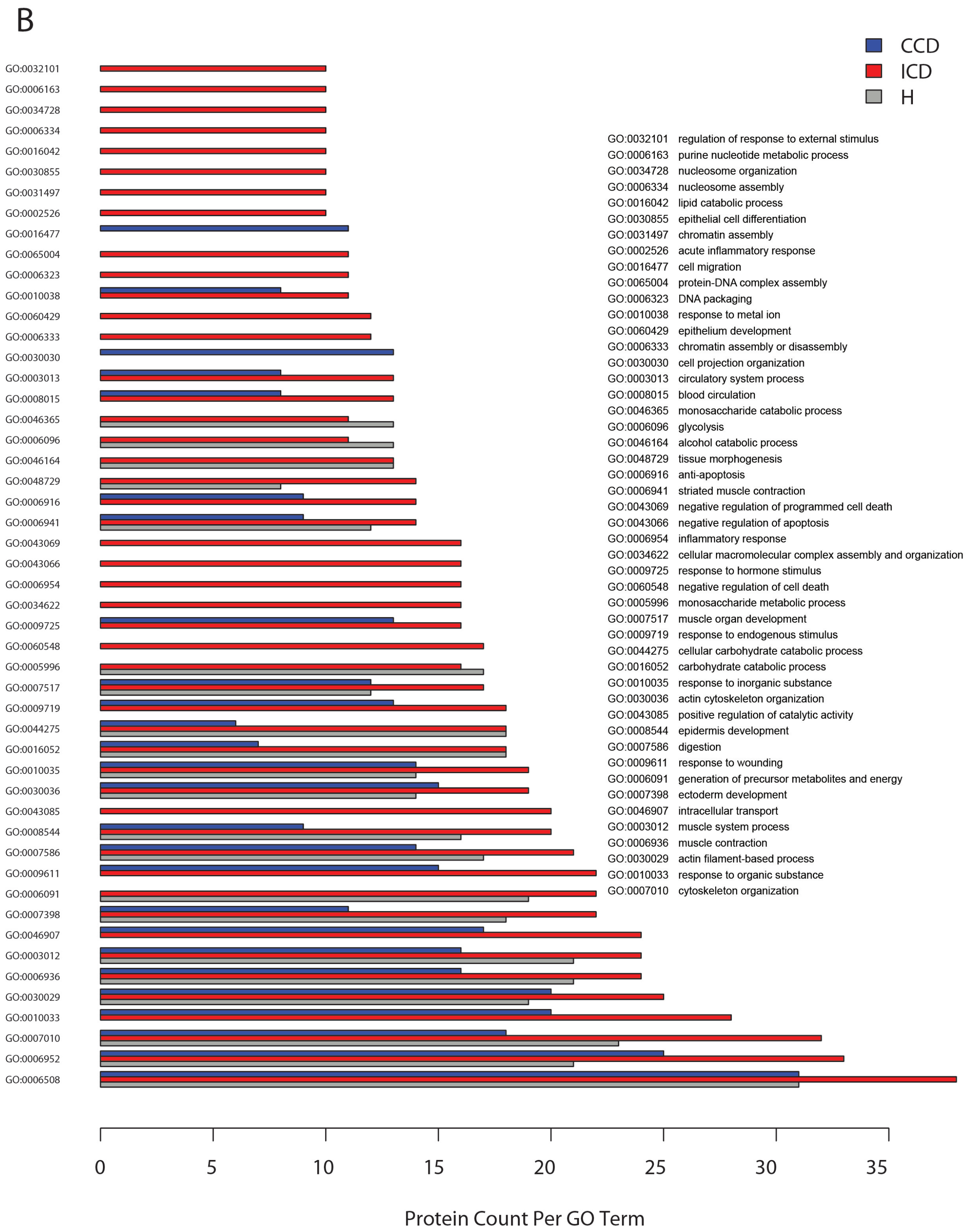
B
CCD
ICD
H
GO:0032101
GO:0006163
GO:0034728
GO:0006334
GO:0016042
GO:0030855
GO:0031497
GO:0002526
GO:0016477
GO:0065004
GO:0006323
GO:0010038
GO:0060429
GO:0006333
GO:0030030
GO:0003013
GO:0008015
GO:0046365
GO:0006096
GO:0046164
GO:0048729
GO:0006916
GO:0006941
GO:0043069
GO:0043066
GO:0006954
GO:0034622
GO:0009725
GO:0060548
GO:0005996
GO:0007517
GO:0009719
GO:0044275
GO:0016052
GO:0010035
GO:0030036
GO:0043085
GO:0008544
GO:0007586
GO:0009611
GO:0006091
GO:0007398
GO:0046907
GO:0003012
GO:0006936
GO:0030029
GO:0010033
GO:0007010
GO:0006952
GO:0006508
GO:0032101 regulation of response to external stimulus
GO:0006163 purine nucleotide metabolic process
GO:0034728 nucleosome organization
GO:0006334 nucleosome assembly
GO:0016042 lipid catabolic process
GO:0030855 epithelial cell differentiation
GO:0031497 chromatin assembly
GO:0002526 acute inflammatory response
GO:0016477 cell migration
GO:0065004 protein-DNA complex assembly
GO:0006323 DNA packaging
GO:0010038 response to metal ion
GO:0060429 epithelium development
GO:0006333 chromatin assembly or disassembly
GO:0030030 cell projection organization
GO:0003013 circulatory system process
GO:0008015 blood circulation
GO:0046365 monosaccharide catabolic process
GO:0006096 glycolysis
GO:0046164 alcohol catabolic process
GO:0048729 tissue morphogenesis
GO:0006916 anti-apoptosis
GO:0006941 striated muscle contraction
GO:0043069 negative regulation of programmed cell death
GO:0043066 negative regulation of apoptosis
GO:0006954 inflammatory response
GO:0034622 cellular macromolecular complex assembly and organization
GO:0009725 response to hormone stimulus
GO:0060548 negative regulation of cell death
GO:0005996 monosaccharide metabolic process
GO:0007517 muscle organ development
GO:0009719 response to endogenous stimulus
GO:0044275 cellular carbohydrate catabolic process
GO:0016052 carbohydrate catabolic process
GO:0010035 response to inorganic substance
GO:0030036 actin cytoskeleton organization
GO:0043085 positive regulation of catalytic activity
GO:0008544 epidermis development
GO:0007586 digestion
GO:0009611 response to wounding
GO:0006091 generation of precursor metabolites and energy
GO:0007398 ectoderm development
GO:0046907 intracellular transport
GO:0003012 muscle system process
GO:0006936 muscle contraction
GO:0030029 actin filament-based process
GO:0010033 response to organic substance
GO:0007010 cytoskeleton organization
0
5
10
15
20
25
30
35
Protein Count Per GO Term

Supporting Tables

Table S1. Twin cohort sample descriptions and details for all subjects, healthy, ileal Crohn's disease (ICD), and colonic Crohn's disease (CCD).

Sample ID	Birth Year	Phenotype	Sex	NOD2 Status	Gastro-enteritis	Age at Diagnosis	Surgery (year)
6a	1951	Healthy	F	nd	Yes	-	-
6b	1951	Healthy	F	nd	No	-	-
9a	1947	CCD, Non-stricturing, Non-penetrating	M	wt	No	41	-
9b	1947	CCD, Non-stricturing, Non-penetrating	M	wt	No	40	-
10a	1962	ICD, Stricturing	F	wt	Yes	23	Ileal res + right hemi (1985)
10b	1962	ICD, Stricturing	F	wt	Yes	24	ileocec res (1986)
15a	1953	ICD, Non-stricturing, Non-penetrating	M	snp 8 m/w	No	23	ileal res (1980)
15b	1953	ICD, Non-stricturing, Non-penetrating	M	snp 8 m/w	No	23	ileocec res (1976)
16a	1954	ICD, Penetrating	F	wt	No	20	ileal res + right hemi (1974)
16b	1954	Healthy Co-twin	F	wt	No	-	-
18a	1953	ICD, Non-stricturing, Non-penetrating	M	wt	No	20	ileal res + right hem (1973)
18b	1953	Healthy Co-twin	M	wt	No	-	-

Abbreviations: ileal res = ileal resection; right hemi = right-sided hemicolectomy; ileal res = ileocecal resection; nd = no data; wt = wildtype.

Table S2. Overview of metagenomic 454 pyrosequencing data.

Subject	Phenotype	Number of BP (M)	Number of Reads (T)	Number of Contigs	BP in Contigs (M)	Average Contig Length (bp)	Number of ORFs
10a	ICD	502	1,211	36,328	49	1337	91,055
10b	ICD	541	1,327	30,642	39	1259	76,707
15a	ICD	504	1,358	38,046	42	1109	80,218
15b	ICD	484	1,270	36,871	36	981	70,548
16a	ICD	229	585	22,764	18	798	43,224
16b	H	319	1,126	46,845	28	591	81,060
18a	ICD	425	1,341	26,803	27	1014	55,030
18b	H	258	955	28,631	19	671	52,512
6a	H	684	1,901	55,650	66	1195	124,910
6b	H	409	1,183	55,463	43	776	106,893
9a	CCD	245	782	19,893	21	1053	41,155
9b	CCD	290	845	35,571	27	754	67,564

Table S3. Proteome metrics (total protein, peptide, and spectra identifications) across all subjects and 24 MS runs for the matched metagenome (MM) database searches.

Phenotype	Sample	Run	Proteins	Peptides	Total Identified MS/MS	Total Collected MS/MS	#DB Entries
healthy	6a	2	2,315	9,110	15,724	78,381	1,356,947
		3	2,009	8,005	15,452	70,492	
	6b	2	1,385	5,548	11,300	80,797	862,006
		1	1,413	5,796	11,258	81,202	
colonic CD	9a	1	871	3,738	7,628	92,865	527,904
		2	829	3,381	7,879	91,745	
	9b	1	723	2,551	5,153	82,546	594,986
		2	728	2,532	5,074	84,089	
ileal CD	10a	3	1,089	4,474	8,891	90,775	1,428,694
		1	1,049	4,286	8,244	91,145	
	10b	1	1,118	4,078	8,057	75,873	1,618,290
		2	1,141	3,984	8,546	74,574	
ileal CD	15a	1	946	3,913	9,276	83,254	1,005,286
		2	1,183	4,862	10,616	77,906	
	15b	2	787	3,890	8,647	81,970	982,092
		1	769	3,421	8,619	80,718	
ileal CD	16a	2	369	1,716	4,079	78,811	1,119,221
		1	407	1,844	4,012	73,878	
healthy	16b	2	1,248	4,581	11,659	92,460	843,556
		3	1,256	4,416	11,914	91,865	
ileal CD	18a	2	687	3,233	7,951	76,489	781,500
		1	654	2,733	7,281	78,661	
healthy	18b	1	794	3,018	7,640	92,795	628,384
		2	829	3,245	7,442	93,485	

Table S4. Proteome metrics (total protein, peptide, and spectra identifications) across all subjects and 24 MS runs for the human microbial isolate reference genome database (HMRG) searches.

Phenotype	Sample	Run	Proteins	Peptides	Total Identified MS/MS	Total Collected MS/MS
healthy	6a	2	3,138	8,679	13,254	78,381
		3	2,618	6,838	11,351	70,492
	6b	2	2,716	6,653	11,022	80,797
		1	2,612	6,308	10,919	81,202
colonic CD	9a	1	2,477	5,910	10,089	92,865
		2	2,337	5,416	10,388	91,745
	9b	1	2,089	5,231	8,485	82,546
		2	2,062	5,161	8,354	84,089
ileal CD	10a	3	2,172	5,997	10,199	90,775
		1	1,985	5,635	9,291	91,145
	10b	1	1,859	4,483	7,964	75,873
		2	1,878	4,071	7,919	74,574
ileal CD	15a	1	1,874	5,494	10,291	83,254
		2	2,122	6,783	11,698	77,906
	15b	2	2,146	6,235	9,816	81,970
		1	1,916	5,430	9,695	80,718
ileal CD	16a	2	1,733	4,738	7,728	78,811
		1	1,840	4,956	7,830	73,878
healthy	16b	2	3,049	6,926	12,610	92,460
		3	3,108	7,070	13,281	91,865
ileal CD	18a	2	1,964	5,931	11,161	76,489
		1	1,648	5,108	10,350	78,661
healthy	18b	1	2,855	6,830	12,669	92,795
		2	3,139	7,626	12,642	93,485

Supporting Note

Metaproteomics Technical and Twin Reproducibility Results

To access the reproducibility of protein quantification by spectra counts, the spectra counts were plotted for technical replicates per subject for all identified proteins for a total of 24 MS technical replicates for 12 subjects. The technical replicates were highly correlated with each other, with the R^2 ranging from 0.881 to 0.988. With the exception of concordant ICD twin pair 10 (R^2=0.669), the metaproteomes of diseased concordant and discordant twins were more variable (R^2 =0.442, 0.513, 0.552, 0.443) for twin pairs 9, 15, 16, and 18, respectively), than those of the healthy twin pair (R^2 =0.657 for twin pair 6).

Metaproteome False Discovery Rates (FDRs)

The peptide-level false discovery rates were estimated using two different approaches for the metaproteomics data from the two different database searches (MM and HMRG) for one representative sample from each category, healthy (6b, run 1), ICD (18a, run 2), and CCD (9a, run 2). As reported in Verberkmoes *et al.* 2009, the false discovery rates were very comparable between both healthy subjects (6a and 6b) and MS runs of which are also used in this study. Previously, Wilkins *et al*. suggested that false discovery rates can hardly ever be accurately estimated with complex metaproteomes datasets due to the level of peptide and protein redundancy seen in microbial communities and their associated databases where an FDR calculated on all peptides can be underestimated whereas as FDR estimated only with unique peptide sequences can be overestimated (Wilkins et al., 2009). Therefore, we calculated FDRs using both approaches; all peptides (redundant) and only unique peptides (non-redundant). The first FDR estimation took into account all identified peptide-spectrum matches (PSMs)

regardless of peptide degeneracy (peptide sequences that are shared by multiple spectra) and is referred to as the "redundant" FDR. On the contrary, the second FDR estimation was based upon using unique peptides only (i.e., PSMs that share an identical peptide sequence are collapsed and represented as one peptide) and is referred to as the "non-redundant" FDR. In addition, an FDR was calculated at a ppm level (±10ppm) for the MM database search results due to the initial liberal ≥1 peptide/read filter that was applied prior to post-database mapping of high mass accuracy peptide-based reads to proteins (≥2 peptide/protein filter).

The "redundant" FDRs for the matched metagenomic database (MM) searches prior to post-database mapping of read-based peptides to assembled contigs (1-peptide level) was calculated as 16.09%, 25.28%, and 30.96% for healthy, ICD, and CCD, respectively, and with high mass accuracy, 1.17%, 1.60%, and 2.36% for healthy, ICD, and CCD. The "non-redundant" FDRs for the matched metagenomic database (MM) searches prior to post-database mapping of read-based peptides to assembled contigs (i.e., ≥1 peptide/read) was 40.67%, 55.87%, and 63.65%, for healthy, ICD, and CCD, respectively, and with high mass accuracy, the FDRs decreased to 2.78%, 3.61%, and 4.66% for healthy, ICD, and CCD (Table S12). Finally, post-database mapping of only the high mass accuracy, non-redundant read-based peptides to assembled contigs (at ≥ 2 peptide/protein), resulted in an FDR of 0.33% for healthy and 0% for both ICD, and CCD.

The "redundant" FDRs for the HMRG database searches was calculated as 0.72%, 0.77%, and 0.97% for healthy, ICD, and CCD, respectively. The "non-redundant" FDRs for the HMRG searches (≥2 peptide/protein) was calculated as 1.31%, 1.33%, and 2.05% for healthy, ICD, and CCD, respectively (Table S11).
